# Lactate and base deficit are predictors of mortality in critically ill patients with cancer

**DOI:** 10.1186/cc10166

**Published:** 2011-06-22

**Authors:** LA Hajjar, JL Vincent, FRBG Galas, JP Almeida, FB Jatene, PC Bueno, JT Fukushima, RE Nakamura, CM Silva, R Kalil Filho, JOC Auler

**Affiliations:** 1 ICESP - FMUSP, São Paulo - SP, Brazil

## Objective

Cancer patients frequently require admission to the intensive care unit (ICU); however, there are few data regarding predictive factors for mortality. The aim of this study was to evaluate whether arterial lactate or standard base deficit (SBD) on admission and after 24 hours can predict ICU and hospital mortality for patients with cancer.

## Methods

We evaluated 1,129 patients with severe sepsis, septic shock, or postoperative after high-risk surgery. Lactate and SBD collected at admission and after 24 hours were compared between survivors and nonsurvivors. We evaluated whether arterial lactate and SBD are independent predictors of ICU and hospital mortality.

## Results

There were 854 hospital survivors (76.5%). Twenty-four-hour lactate >1.9 mmol/l (OR = 4.02, CI = 2.7 to 5.97) and SBD <-2.3 (OR = 2.4, CI = 1.64 to 3.52) were independent predictors of ICU mortality. Twenty-four-hour lactate >1.9 mmol/l (HR = 2.63, CI = 1.99 to 3.47) and 24-hour SBD <-2.3 mmol/l (HR = 1.74, CI = 1.33 to 2.27) were independent predictors of hospital death.

## Conclusion

Our findings suggest that lactate and SBD measurement should be included in the routine assessment of patients with cancer admitted to the ICU. These markers may be useful in the adequate allocation of resources in this population.

